# Bruton's tyrosine kinase regulates TLR7/8-induced TNF transcription via nuclear factor-κB recruitment

**DOI:** 10.1016/j.bbrc.2018.03.140

**Published:** 2018-05-05

**Authors:** Theresa H. Page, Anna M. Urbaniak, Ana I. Espirito Santo, Lynett Danks, Timothy Smallie, Lynn M. Williams, Nicole J. Horwood

**Affiliations:** Kennedy Institute of Rheumatology, NDORMS, University of Oxford, Roosevelt Drive, Oxford, OX3 7FY, United Kingdom

**Keywords:** Macrophages, Bruton's tyrosine kinase, Toll-like receptors-7/8, R848, NFκB

## Abstract

Tumour necrosis factor (TNF) is produced by primary human macrophages in response to stimulation by exogenous pathogen-associated molecular patterns (PAMPs) and endogenous damage-associated molecular patterns (DAMPs) via Toll-like receptor (TLR) signalling. However, uncontrolled TNF production can be deleterious and hence it is tightly controlled at multiple stages. We have previously shown that Bruton's tyrosine kinase (Btk) regulates TLR4-induced TNF production via p38 MAP Kinase by stabilising TNF messenger RNA. Using both gene over-expression and siRNA-mediated knockdown we have examined the role of Btk in TLR7/8 mediated TNF production. Our data shows that Btk acts in the TLR7/8 pathway and mediates Ser-536 phosphorylation of p65 RelA and subsequent nuclear entry in primary human macrophages. These data show an important role for Btk in TLR7/8 mediated TNF production and reveal distinct differences for Btk in TLR4 versus TLR7/8 signalling.

## Highlights

• Btk is required for TLR7/8 signalling in primary human macrophages.
• R848-induced TNF mRNA is more Btk dependent than LPS-induced TNF mRNA.
• Btk transcriptional control of TNF following R848 requires the promoter and 3′UTR.
• Btk knockdown reduces p65RelA translocation to the nucleus upon TLR7/8 stimulation.

## 1 Introduction

TNF production is precisely regulated at both the gene and protein expression level [1]. Toll-like receptors (TLRs), by recognising ligands as diverse as bacterial cell wall components and nucleic acids, are important inducers of TNF production in disease. In addition, recognition of endogenously derived damage-associated molecular patterns (DAMPs) makes them key players in the induction and maintenance of autoimmune inflammation [2].

Non-receptor tyrosine kinases play a major role in TLR signalling [[3], [4], [5]], and in particular, Bruton's Tyrosine Kinase (Btk), a member of the Tec family of non-receptor protein tyrosine kinases (PTKs), is a crucial regulator of TLR induced TNF production [6,7]. In humans, a lack of functional Btk leads to X-linked agammaglobulinemia (XLA), a condition characterised by both B cell deficiency and ineffective immune responses to bacterial and viral challenge [8]. XLA patient monocytes show reduced production of TNF and IL-1β in response to TLR2 and TLR4 ligands [9,10] and stimulation of XLA-derived dendritic cells with siRNA results in significantly decreased production of both TNF and IL-6 [11]. Btk deficiency in B cells reduces TLR9-induced production of IL-10, leading to elevated levels of TNF, IL-6 and IL-12p40 [12,13] a finding that may explain the increased levels of cytokines present in XLA serum [14].

In HEK293 cells Btk physically interacts with the cytoplasmic Toll/IL-1 receptor (TIR) domains of TLRs 4, 6, 8 and 9 as well as the adaptor molecules Myd88 and Myd88-adapter-like (Mal) [15]. Following stimulation, TLR receptors (except TLR3) recruit Myd88 via its cytoplasmic Toll/IL-1 receptor (TIR) domain. Various other molecules including IL-1 receptor-associated kinases 1 and 4 (IRAKs 1 and 4), TNF receptor associated factor (TRAF) 6, TAB2/3 and TAK1 then associate with the receptor complex. IκB is phosphorylated by the TAK1-activated IκB kinase (IKK) complex, ubiquitinated and degraded by the 26S proteasome. Following NFκB release from the inhibitory IκB complex, p65RelA is phosphorylated on a number of serines to regulate p65RelA nuclear translocation and gene transactivation [16]. NFκB is considered to be essential for TNF transcription, and over-expression of IκBα decreases TNF production from LPS-stimulated human primary macrophages [17].

Here we provide evidence for Btk in TLR7/8 signalling in human primary macrophages. Btk regulates TLR7/8-induced TNF production at early time points via the 3'enhancer region of the TNF gene. Moreover, we show that Btk controls the initiation of TNF transcription through NF-κB recruitment. Interestingly, TNF transcription in response to LPS was less affected, revealing a previously unreported distinction between TLR4 and TLR7/8-mediated TNF gene transcription.

## 2 Materials and methods

*Reagents and Antibodies*. R848 and LPS were from Alexis Biochemicals and macrophage colony-stimulating factor (M-CSF) was from Peprotech. Polyclonal rabbit anti-Btk antibody for immunoprecipitation was a gift from M. Tomlinson (University of Birmingham, U.K.), mouse anti-Btk antibody (clone 10D11) for Western blotting was from BD Bioscience and anti-GAPDH (ab9484) and rabbit isotope control antibodies were from Abcam. The phosphotyrosine clone 4G10 was from Millipore. Cell Signaling provided anti-IκBα (#9242), anti-phospho-p65RelA (Ser536) (for Western blot; #3036), and anti-p65RelA (for confocal; #3033). Anti-p65RelA (sc-372) for Western blotting was from Santa Cruz Biotechnology.

*Monocyte isolation and adenoviral infection*. Following ficoll-hypaque centrifugation, monocytes were elutriated from PBMC as previously described [17]. Monocytes were treated with M-CSF (100 ng/ml) for 3–4 days prior to counting and re-seeding. Creation of adenoviral constructs and method of infection as previously described [17]. For double infections, cells were first infected with the luciferase adenovirus at multiplicity of infection (moi) 50:1 for 2h, rested for 4h in serum containing medium prior to secondary infection at moi 100. Luciferase reporter assays were performed as previously described [9,18].

*Gene knockdown by siRNA*. 5 × 10^6^ primary human monocytes were transfected with targeting siRNA or control oligunucleotides (siControl D-001206-13 and human Btk SMARTpool M-003107-01, Dharmacon, IL) at concentrations ranging from 100 to 300 nM using Human Monocyte Nucleofector Kit (Amaxa Biosystems, Germany) according to manufacturer's instructions. After 24 h, monocytes were cultured in 5% HIFCS phenol red free RPMI with 100 ng/ml M-CSF for a further 72h. STAT1 phosphorylation by Western blot following siRNA nucleofection was assessed after a further 24h in the absence of M-CSF and stimulation ± IFN (1 ng/ml) for 5 min.

*Immunoprecipitation and Western Blotting*. M-CSF-differentiated macrophages were plated on 10 cm^2^ petri dishes and serum starved for 2h prior to stimulation. Cells were lysed in ice-cold lysis buffer (20 mM Tris-Base pH 7.6125 mM NaCl, and 1% Nonidet P-40), containing freshly added 10 mM DTT, 100 μM Na_2_VO_3_, 5 mM NaF, 1x Protein Inhibitor Cocktail (Sigma). Debris was removed by centrifugation, and supernatants were pre-cleared with protein G-sepharose. Btk was precipitated with polyclonal rabbit anti-Btk anti-sera and protein G-sepharose for 1.5 h. Immunoprecipitated complexes were washed in lysis buffer before resolving on 10% SDS-PAGE gel and transferring to nitrocellulose membrane (Millipore). The membrane was blocked for 1h in TBS-Tween (0.1%) with 2% BSA for the detection of phosphorylated proteins or in 5% skimmed milk for other proteins.

*Immunocytochemistry.* After siRNA transfection, monocytes were differentiated in M-CSF for 72h. Macrophages were plated on glass coverslips (ECN 631-1578, VWR) and stimulated with R848 (1 μg/ml). Cells were fixed with 4% (w/v) paraformaldehyde in PBS for 15 min at 37 °C, quenched with 50 mM NH_4_Cl/PBS for 10 min, and permeabilised with 0.1% (w/v) Triton X-100 in PBS for 5 min. Samples were blocked with 3% (w/v) BSA in PBS for 30 min at room temperature followed by incubation with anti-phospho-p65RelA (Ser536) diluted in 3% (w/v) BSA in PBS for 1 h at room temperature. After washing, secondary antibody was added; phosphorylated p65RelA (Ser536) with Alexa Fluor 488 (A11034, Invitrogen), actin cytoskeleton with Alexa Fluor 546 Phalloidin (A22283, Invitrogen) and the nucleus with DAPI (D1306, Invitrogen). Samples were mounted with ProGold antifade mounting media (P36934, Invitrogen). Confocal ‘z’ stack were used to quantify the intensity of staining by measuring the respective brightness of the pixels for each of detection channels using Fiji image analysis software.

*Cytokine measurements by ELISA*. TNF concentration in supernatants was determined by ELISA (BD Biosciences), according to the manufacturer's instructions. Absorbance was read and analysed at 450 nm using a Fluostar Omega (BMG Labtech, Aylesbury, UK) plate reader and analysed using MARS data analysis software.

*Real-time RT-PCR*. RNA was extracted from macrophages using Blood RNA extraction kit (QIAGEN), and genomic DNA removed using TURBO DNA-free kit (Applied Biosystems). cDNA was subjected to real-time PCR analysis using SYBR Premix Ex Taq (Lonza) on a Corbett Rotor-Gene 6000 (Qiagen). Primers for measuring primary human TNF transcripts were 5′-GCAGTCAGATCATCTTCTCG-3′ and 5′-GGTACAGGCCCTCTGATGGCAC-3’. Mature human TNF transcripts were 5′-CCTGCTGCACTTTGGAGTGATCGG-3’ & 5′-GTACAGGCCCTCTGATGGCACCACC-3′, respectively. Primers for actin-related protein transcripts (ARP) were 5′- CGACCTGGAAGTCCAACTAC-3′ and 5′- ATCTGCTGCATCTGCTTG-3’. Relative quantification of gene expression was expressed as fold mRNA/ARP as determined using the comparative ΔΔCT method.

*Statistical analysis*. Values correspond to mean ± SEM or SD. In experiments with multiple groups, differences were first evaluated using repeated-measures ANOVA and then Dunnett's test to compare group means. Unpaired Student's *t*-test was used when comparing differences between two groups.

## 3 Results

### 3.1 TNF production following TLR7/8 stimulation requires Btk

**Fig. 1:**
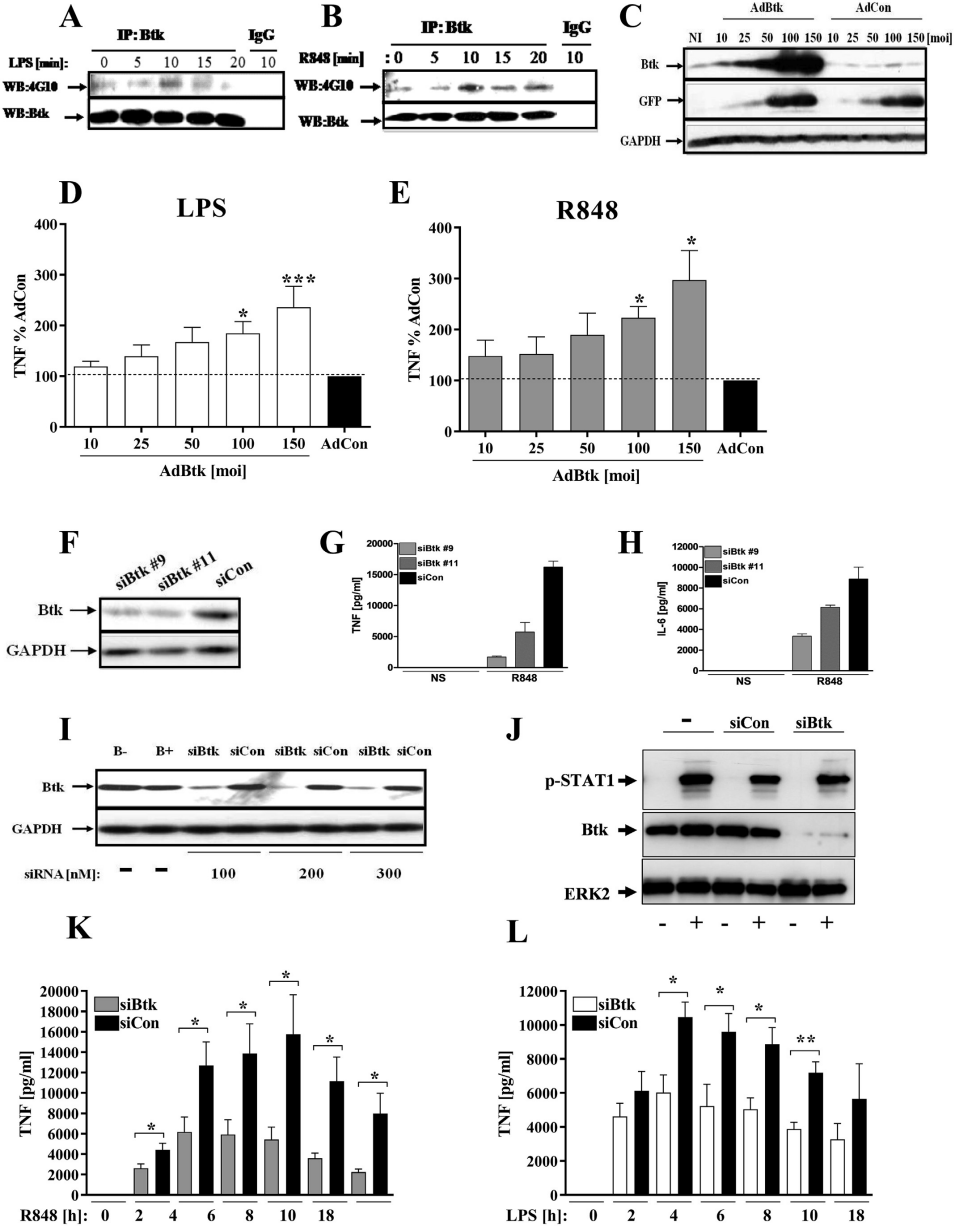
*Btk is required for cytokine production in TLR4 and TLR7/8 signalling*. M-CSF-differentiated macrophages were (A, B) serum starved for 2h prior to stimulation with 10 ng/ml LPS or 1 μg/ml R848 up to 20 min. Btk was immunoprecipitated using anti-Btk antibody followed by western blotting with 4G10 antibody. Anti-rabbit IgG as isotope control. (C) Macrophages infected with adenovirus at moi 10 to 150 were left for 48 h. Btk expression in cell lysates by Western blot using GAPDH as a protein loading control. (D, E) Following infection, cells were stimulated with either LPS (10 ng/ml) or R848 (1 μg/ml) for 18h and TNF production assessed by ELISA. Btk over-expression was normalised to control (black bar; empty adenovirus) for each moi. (F) Monocytes transfected with Btk siRNA (siBtk#9 and #11) or control siRNA (siCon) at 200 nM then M-CSF-treated for 4 days and Btk expression assessed by Western blot. (G, H) TNF or IL-6 in supernatants was measured by ELISA from siRNA manipulated cells after 18hr with either no stimulus or 1ug/ml R848. (I) Monocytes were transfected with Btk siRNA (siBtk) or control siRNA (siCon) at 100, 200 or 300 nM and then M-CSF-treated for 4 days. B- was no transfection; B+ transfection with buffer alone. Cell lysate expression of Btk was assessed by Western blot. (J) Monocytes were incubated with no oligo (nil), control oligo or BTK oligo at 200 nM prior to nucleofection. After 24 h of M-CSF treatment, media was replaced, and after a further 24 h cells were plated and stimulated ± IFN (1 ng/ml) for 5 min. Cell lysates were used to determine the phosphorylation status of STAT1 and levels of BTK by SDS-PAGE and western blotting. (K, L) Monocytes were transfected with 200 nM siBtk or siCon, M-CSF-treated for 4 days, and then stimulated with either LPS (10 ng/ml) or R848 (1 μg/ml) for 18h; TNF concentration was assessed by ELISA. Cytokine levels are expressed as means of triplicate repeats ± SEM. Results shown combined data from 3 to 6 separate donors. Statistical analysis: student's t-test, ***p < 0.001, **p < 0.01, *p < 0.05.

Enzymatic activation of Btk is marked by tyrosine phosphorylation. Primary human macrophages were stimulated with either LPS (10 ng/ml) or R848 (1 μg/ml) for 5, 10, 15 or 20min and tyrosine phosphorylation of Btk determined by Western blot analysis. As previously observed [9] an increase in Btk phosphorylation occurred in response to LPS (TLR4) stimulation with a maximal response at 10min (Fig. 1A). Likewise, an increase in Btk phosphorylation occurred within 10mins of R848 (TLR7/8) stimulation (Fig. 1B).

To determine whether Btk modulates TLR7/8 driven TNF, Btk was over-expressed as previously described [9] as confirmed by western blot (Fig. 1C). Cells were then stimulated with either LPS or R848 and TNF production after 18 h was measured. Over-expression of Btk resulted in significantly increased TNF production in response to both LPS and R848 (Fig. 1D and E). To test the effect of Btk down-regulation on TNF production two siRNA duplexes; siBtk#9 and siBtk#11 were tested and produced similar decreases in Btk expression (Fig. 1 F). In the absence of TLR stimuli (NS), siRNA nucleofection alone did not induce any detectable cytokine production (Fig. 1G and H). In the presence of R848, siBtk#9 showed the greatest effect on cytokine production and hence was used for the remaining experiments (Fig. 1G and H). Three doses of siRNA were tested (100, 200 and 300 nM) and 200 nM was chosen as the concentration for subsequent experiments. Additionally, there was no basal increase in STAT1 phosphorylation following RNAi knockdown indicating that subsequent findings are attributable to Btk knockdown as opposed to the induction of an interferon response (Fig. 1J). Following Btk inhibition, TNF release was significantly reduced from 4 to 10h after LPS stimulation and 2–18h after R848 stimulation (Fig. 1K and L). Interestingly the effect of Btk ablation was more pronounced and longer lasting for R848-induced TNF production than for LPS.

### 3.2 Effect of Btk depletion on TLR4-and TLR7/8-mediated TNF transcription

TNF is controlled at a number of different stages in its production. Our previous work showed that message stability is an important control point for TNF production [9]. TNF is also controlled at the transcriptional level and data from other workers in HEK293s and immortalised monocyte lines have suggested that this is how Btk mediates its effect on TLR7/8 induced TNF [19].

**Fig. 2:**
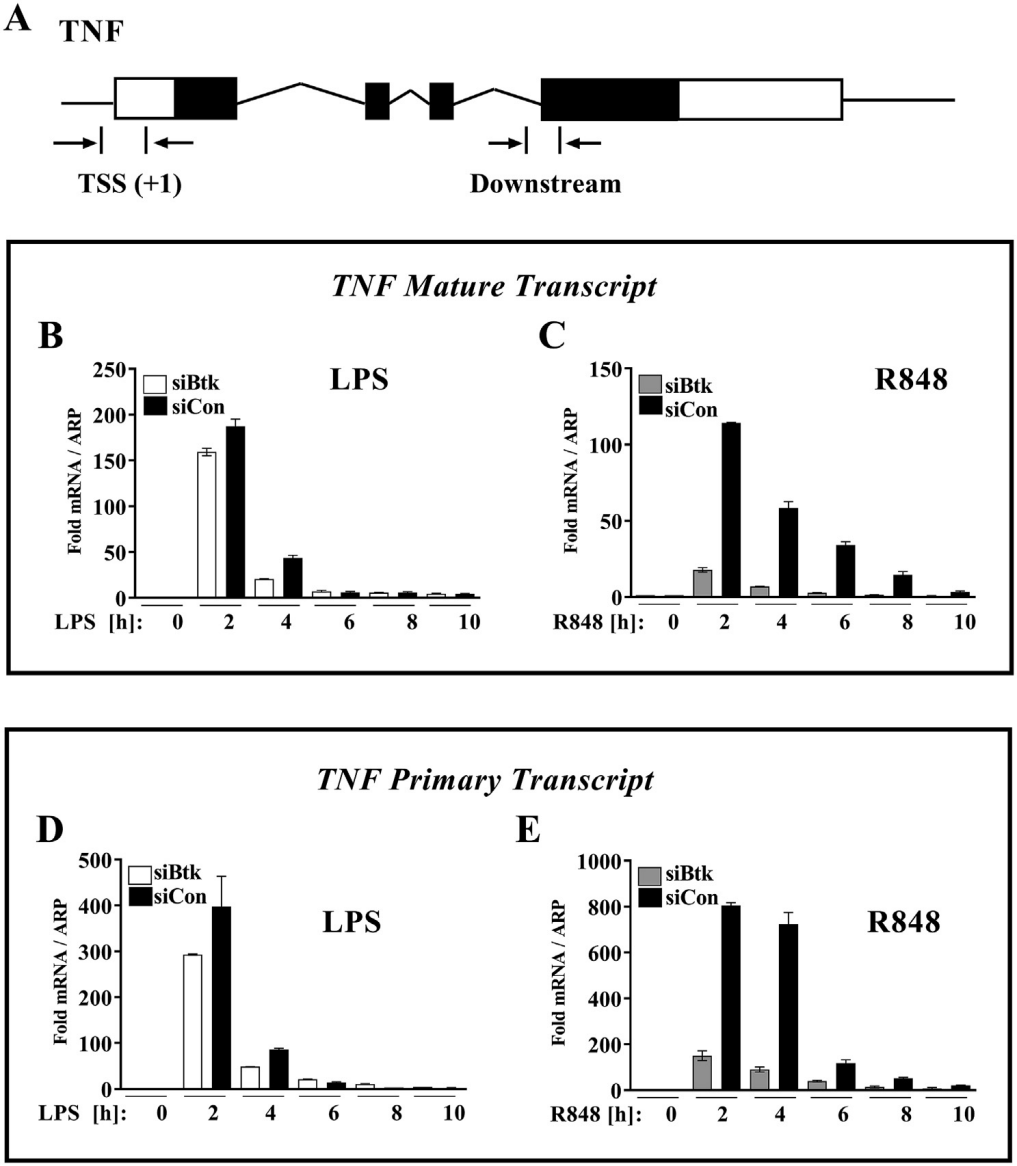
*Btk regulates TLR7/8-induced TNF transcription.* (**A**) Schematic representation of the TNF gene indicating primer pair positions used for transcriptional start site (TSS) and downstream regions of the gene. Monocytes were transfected with 200 nM Btk siRNA (siBtk) or control siRNA (siCon), M-CSF-treated for 4 days, and then stimulated with either LPS (10 ng/ml) (**B and D**) or R848 (1 μg/ml) (**C and E**) up to 10h. TNF mature (**B, C**) and primary (**D, E**) mRNA levels measured by RT-PCR using primers for downstream or TSS regions of the TNF gene. Graphs show mean values ± SD of triplicate measurements for a single donor: representative of 3 independent experiments using different donors.

To establish whether this is also true of primary human cells we examined mature TNF transcripts in macrophages following siBtk. Real-Time PCR with a primer set spanning an exon-exon TNF sequence (Fig. 2A) were used for the detection of mature mRNA [18]. Fig. 2B and C shows that mature TNF mRNA production lasts longer after R848 stimulation with elevated levels still found after 8 h. In comparison, LPS stimulated cells show a more transient induction of TNF message; this may explain why R848 stimulated cell cultures consistently had higher TNF expression levels. Following Btk knockdown LPS-stimulated cells showed approximately 15% decrease at 2 h and no more than a 50% decrease in transcript level at 4 h (Fig. 2B), whilst in R848-stimulated cells mature TNF transcripts were decreased by at least 80% at all of the time points (Fig. 2C).

In response to Btk knockdown, primary TNF transcripts (analysed using primers over an intron-exon boundary of TNF; Fig. 2A) showed a similar pattern demonstrating that the reduction in transcript level is due to deficient transcription rather than RNA processing (Fig. 2D and E). Taken together, these data reveal fundamental differences between the way that Btk controls TNF production downstream of TLR4 and TLR7/8 stimulation with R848-induced TNF mRNA being more Btk dependent than that induced by LPS.

### 3.3 NF-κB sites in TNF promoter and a 252bp region of TNF 3′UTR are required for Btk effects

Transcription of TNF is mediated by a number of transcription factors including AP-1, Oct-1 and NF-κB [20]. 4 distinct NF-κB binding regions have been described within the *TNF* locus; 3 sites (κB1, κB2/ζ/2a, κB3) within the promoter region and a fourth cluster (sites 4, 4a and 4b) in the 3′UTR. Site 4 plays an essential role in LPS-mediated TNF transcription in macrophages and DCs [18,21] and our previous studies have highlighted the importance of a 252bp region in the 3′UTR of TNF for TLR4-induced transcription [18].

**Fig. 3:**
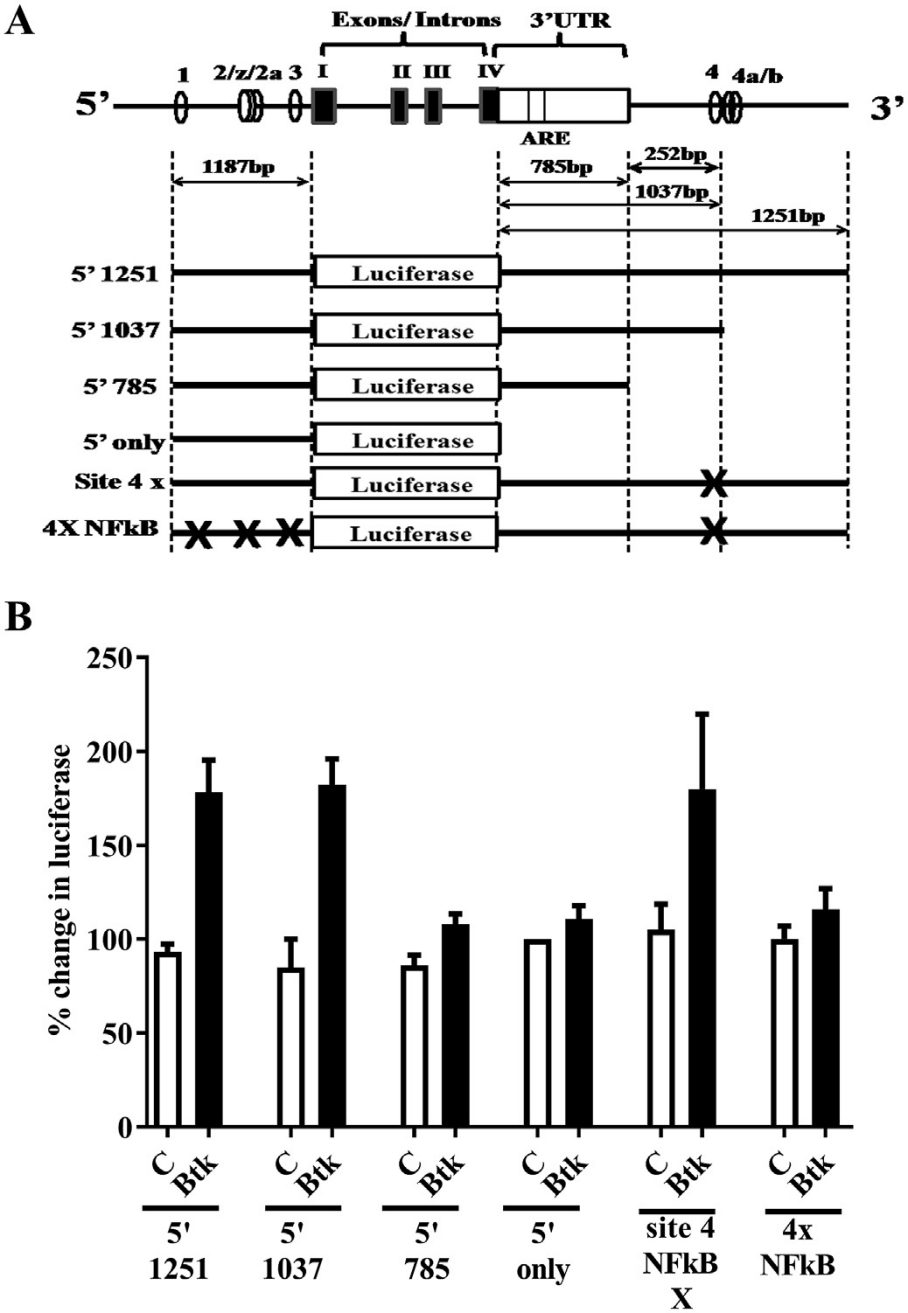
*Btk regulates TLR7/8-induced TNF via both promoter NFkB sites and downstream regions of the gene.* (**A**) Adenoviral luciferase reporters based on the human TNF gene. Positions of NFκB sites (2/ζ/2a, 3 and 4/4a/b) are shown. AAA denotes site of poly-A tail. X indicates position of point mutations used to destroy NFκB site(s). (**B**) M-CSF-differentiated macrophages were infected with luciferase reporter adenovirus at moi 50 followed by AdBtk or AdCon at moi 100. After 24h cells were stimulated with R848 (1 μg/ml) and lysed after 6h. Values are normalised to non-infected controls and represent combined data from >3 separate donors (means ± SEM).

To investigate the NF-κB sites, and other regions in the TNF locus, controlling Btk-mediated TLR7/8-induced transcription we used a series of adenoviral constructs containing the TNF promoter, 3′UTR and other downstream regions, linked to a luciferase reporter (Fig. 3A). Over-expression of Btk produced a 2-fold increase in TLR7/8 driven luciferase production in the 5′1251 construct containing the full-length promoter (1187bp) and downstream sequences (1251bp) (Fig. 3B). Luciferase production by the 5′1037 construct (lacking the terminal 214bp and NF-κB sites 4a and 4b) and the ‘site4X’ construct (includes a mutation that inactivates NF-κB site 4), also show a 2-fold increase in luciferase production indicating that neither the terminal 214bp nor the site 4 NF-κB cluster are required for Btk to influence TLR7/8-induced TNF production.

In contrast, the 5'only construct, the 4X NF-κB construct (all NF-κB sites destroyed by point mutations), and the 5′785 construct, which lacks a 252bp region in the 3′UTR, were not able to increase luciferase production after Btk over-expression (Fig. 3B). Thus demonstrating the need for both the NF-κB sites in the TNF promoter (site 1, cluster 2, site 3), and the 252bp region in the 3′UTR to mediate the effect of Btk on TLR7/8 driven TNF production.

### 3.4 Btk mediates phosphorylation and nuclear entry of p65RelA in response to TLR7/8

**Fig. 4:**
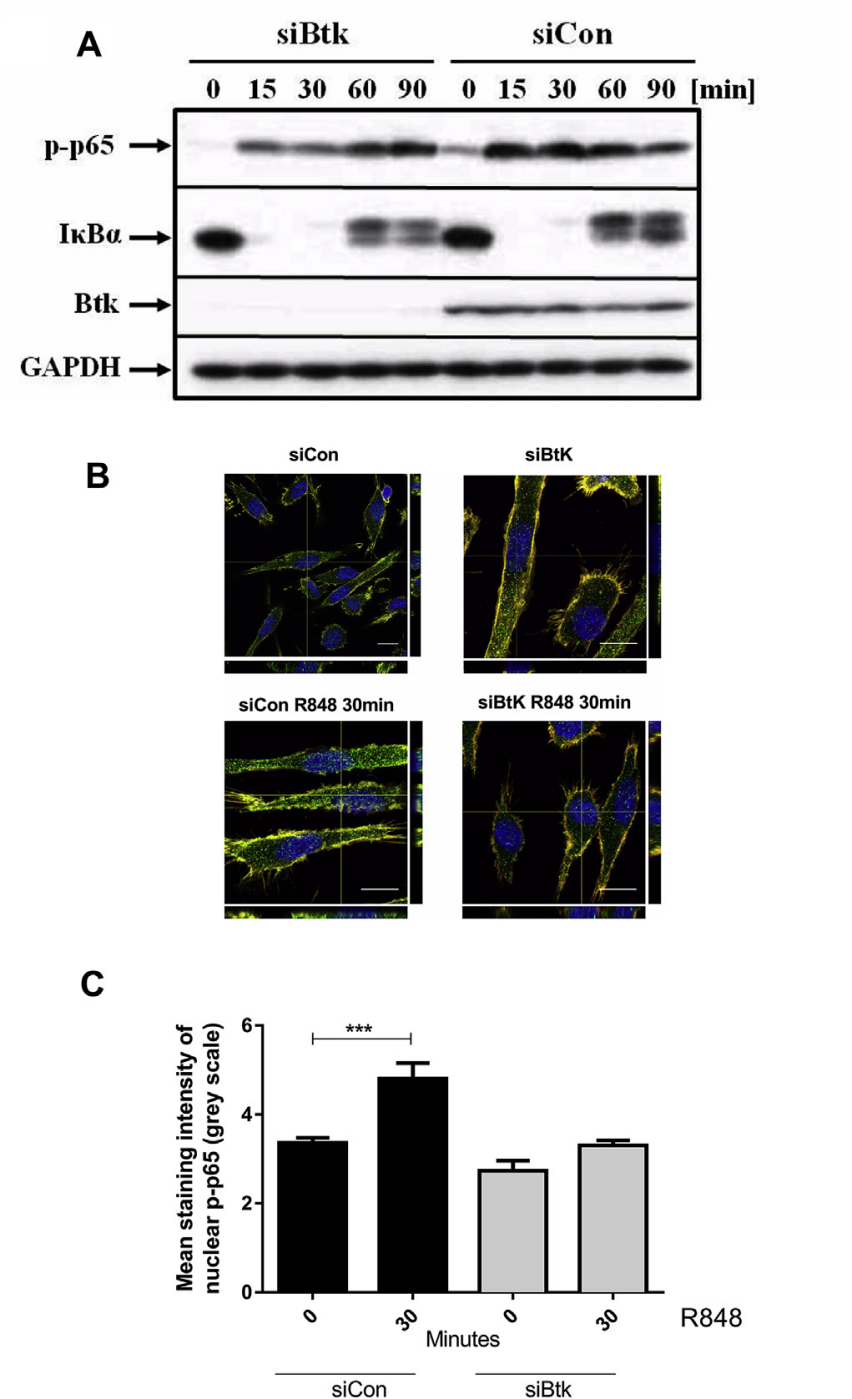
*Btk down-regulation decreases R848-induced p65 phosphorylation.* (**A**) Monocytes were transfected with 200 nM Btk siRNA (siBtk) or control siRNA (siCon), M-CSF-treated for 4 days, and then stimulated with R848 (1 μg/ml) for 90min. p65 RelA phosphorylation and degradation of IκBα was assessed by western blotting with anti-p-p65 (Ser536) and anti-IκBα antibodies. Blot represents one of 3 independent experiments. (**B**) siRNA transfection of monocytes was performed as described in (A). Primary human macrophages were plated on glass coverslips, stimulated with R848 (1 μg/ml) for 30min, fixed and stained for phosphorylated p65RelA (green), actin (red) and nucleus (blue) (scale bars = 10 μm). Images are representative of 3 independent experiments. (**C**) Staining intensity of p-p65 in the nucleus. Mean values from 2 to 3 nuclei per each condition, for each of 3 independent experiments (means ± SEM). Statistical analysis: student's t-test. ***p = 0.0003. (For interpretation of the references to colour in this figure legend, the reader is referred to the Web version of this article.)

Studies to determine the effect of Btk on TLR7/8 mediated IκBα activity showed that knockdown of Btk had no effect TLR7/8-mediated phosphorylation and degradation of IκBα (Fig. 4A). However, NF-κB activity can be regulated at many levels. In particular, p65RelA phosphorylation on Ser536 is described to be independent of IκBα activity and to be necessary for p65RelA nuclear entry. After Btk knockdown, confocal microscopy experiments showed a significant reduction (***p = 0.0003) in p65RelA translocation into the nucleus in human macrophages upon TLR7/8 stimulation (Fig. 4B and C).

## 4 Discussion

Understanding how TNF expression is controlled is crucial for the next generation of anti-inflammatory therapies [22]. TLR7 and 8 both recognise single stranded RNA, which is central to the recognition of both viral and bacterial pathogens [23,24]. The ability of TLR7/8 to recognise endogenously released RNA has implications for autoimmune diseases where TLR7/8 contributes to inflammation in RA and SLE [25,26].

Btk is a critical signalling component of a wide variety of immune receptors, including Fc receptors, gp130 containing cytokine receptors, and the B cell receptor where it is required for the activation of NF-κB [6,27]; the cumulative effect of Btk depletion is an impaired immune response. Downstream of TLR7/8 engagement numerous signalling pathways are activated, including the NF-κB, MAPK, AP1 and the IRF family [23,28]. Doyle et al. showed that TLR8 stimulation activates Btk in THP1 cells via phosphorylation of tyrosine 223 and increased autokinase activity [19]. TLR8-mediated Ser536 phosphorylation of p65RelA in murine BMDM was significantly reduced in Xid mice [19]; our data agrees with these findings and extends them to show that TLR7/8 mediated activation of Btk occurs in primary human macrophages. Additionally, we reveal distinct differences in the requirement for Btk in TLR4 and TLR7/8 signalling within the same cell type. In TLR7/8 signalling Btk acts upstream of NF-κB activation, regulating the serine phosphorylation of p65RelA and thereby its nuclear localisation - consequently, TNF transcription is significantly inhibited without Btk. In contrast, despite similar reductions in p65RelA binding to NF-κB sites in Btk depleted cells after LPS (TLR4) stimulation, transcription of TNF was largely un-affected. Btk-independent TNF transcription following LPS suggests that additional transcription factors/regulatory mechanisms may be active for TLR4 signalling that are absent for TLR7/8.

Phosphorylation of p65RelA occurs after release from the inhibitory IκBα complex and the importance of Ser536 phosphorylation of p65RelA is demonstrated by the mutation of Ser536 to alanine which abolishes the interaction of p65RelA with the transcriptional coactivators p300 and CREB-binding protein (CBP) thereby decreasing transcriptional activation [29,30]. Btk lies upstream of this phosphorylation event in a number of systems, including signalling via TLR4 and the B cell receptor [30,31], but the mechanism is unknown. p65RelA can be phosphorylated on Ser536 by IKKα and IKKβ for example, but neither molecule has been described to interact with Btk.

Non-receptor tyrosine kinases are attractive targets for therapeutic intervention as small molecule inhibitors are readily synthesised [32]. Btk inhibitors have demonstrated considerable success in clinical trials, particularly in combating B cell malignancies [33,34] and in the treatment of autoimmune disorders such as lupus and inflammatory arthritis in animal models [[35], [36], [37]]. Btk inhibition acts not only via reduced B cell development and function, but also on other cell types such macrophages limiting cytokine and chemokine production [38,39].

We have used loss of function and overexpression manipulation technologies to demonstrate the importance of Btk in response to different TLR ligands in primary human macrophages. Btk plays an important role in the TLR7/8 mediated induction of TNF and there are clear differences between TLR4 and TLR7/8-mediated TNF production. These data add important insight into the control of TNF production in primary human cells and may suggest molecular targets for the development of more efficient anti-inflammatory therapeutics.
